# Coupling Effects of Depression and Functional Disability: Using Bivariate Latent Change Score Model

**DOI:** 10.1093/geroni/igab046.3376

**Published:** 2021-12-17

**Authors:** Gina Lee, Peter Martin

**Affiliations:** Iowa State University, Ames, Iowa, United States

## Abstract

The purpose of the study was to examine the coupling effect of depression and functional disability over four time points using the data from the Health and Retirement Study (HRS). The sample included participants who survived to 98 years or older (N = 458). Four alternative latent change score models were computed to examine the univariate and bivariate effects among depressive symptoms (CES-D) and functional disabilities (ADL): No-coupling, univariate model of ADL to change in CES-D, univariate model of CES-D to change in ADL, and bivariate model. As hypothesized, the no-coupling model did not fit the data well, χ2 (26) = 164.86, CFI = 0.85, RMSEA = 0.11. Model 2, ADL predicting change in CES-D, did not fit the data well, χ2 (25) = 164.18, CFI = 0.85, RMSEA = 0.11. Model 3, CES-D predicting change in ADL, also did not fit the data, χ2 (25) = 148.06, CFI = 0.87, RMSEA = 0.10. The bivariate model fit the data well, χ2 (21) = 66.94, CFI = 0.95, RMSEA = 0.07, and was the best fitting model. All level to change effects were significant in model 4. One’s CES-D at prior waves was positively associated with change in ADL at subsequent waves, and ADL at prior waves was positively associated with change in CES-D at subsequent waves. In conclusion, there is a significant coupling effect between depressive symptoms and ADL over time. Future health policies should monitor older adults’ mental and functional health simultaneously for their possible spillover effects.

